# Supplementary material for: Modulation of carbon-to-nitrogen ratio shapes the microbial ecology in a methanol-fed recirculating marine denitrifying reactor
Source: PeerJ. 2025 Oct 13;13:e20129. doi: 10.7717/peerj.20129 (PMC12530212; doi:10.7717/peerj.20129)
Supplement: Supplemental Information 1 [file peerj-13-20129-s001.pdf]

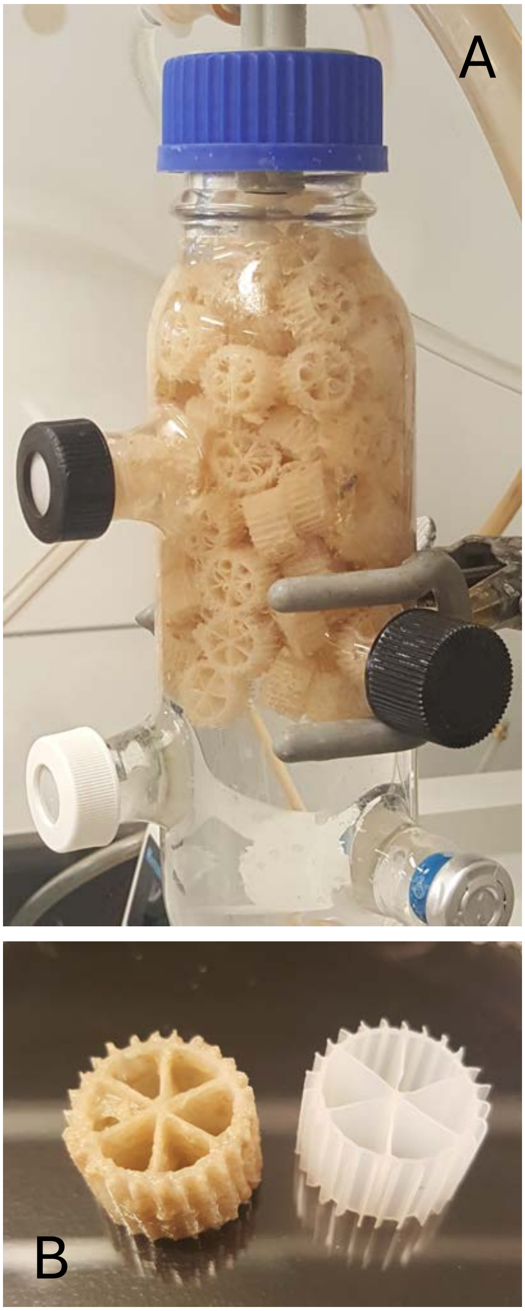

Figure S1: Biofilm on the reactor supports

Panel A. Recirculating reactor

Panel B. Left: support with biofilm. Right: virgin support
