# Supplementary material for: Modulation of carbon-to-nitrogen ratio shapes the microbial ecology in a methanol-fed recirculating marine denitrifying reactor
Source: PeerJ. 2025 Oct 13;13:e20129. doi: 10.7717/peerj.20129 (PMC12530212; doi:10.7717/peerj.20129)
Supplement: Supplemental Information 7 [file peerj-13-20129-s007.docx]

**Supplemental document #6**

**Title**

Influence of carbon-to-nitrogen ratio on the denitrifying activities of a methanol-fed, marine recirculating denitrification reactor, and the evolution of the bacteria community during the operating process.

**Authors**

Livie Lestin^1^, Richard Villemur^1*^

^1^ Institut national de la recherche scientifique (INRS)

Centre Armand-Frappier Santé Biotechnologie

Laval, Province of Quebec, Canada, H7V 1B7

***Correspondence**

Corresponding Author: Richard Villemur

Email address: [richard.villemur@inrs.ca](mailto:richard.villemur@inrs.ca)

**Process of the metatranscriptomic reads**

Sequencing reads for the metatranscriptomes were first aligned to the genomes and plasmids of *M. nitratireducenticrescens* strain GP59 and strain JAM1, and of *Hyphomicrobium nitrativorans* strain NL23. Between 2.7% to 8.5% reads aligned to these genomes in the five biofilm samples (Table S1). Reads that did not align were *de novo* assembled, and the relative transcript levels of the resulting contigs was determined by aligning reads to them. We designated these relative values as TPM-RSEM (method of quantitative alignment). The contigs were analyzed for the presence of open reading frame (ORF) and processed to databases to determine their putative function and their most probable taxon affiliation. Among contigs that contain ORF, between 4.8% to 20.3% of TPM-RSEM were associated to these ORF, most of them (> 90%) are affiliated to Bacterial lineages (Table S1). The contigs were analyzed for the presence of ORF and processed to databases to determine their putative function and their most probable taxon affiliation (Table S2). The remaining TPM-RSEM values were associated with ncRNA (Table S1) and described and discussed below.

**Table S1. Proportion of transcriptomic reads affiliated to lineage or to ncRNA**

OB C1-1 C4-1 C6-1 C8-1

Total reads 56 395 484 61 055 437 56 507 228 57 224 127 56 982 986

**% reads associated to strains GP59, JAM1 and NL23***

NL23 +JAM1 0.95 0.28 0.22 0.35 0.34

GP59 1.78 6.45 8.24 7.89 6.02

**Proportion (TPM-RSEM) of unaligned reads associated to ORFs and their affiliation****

Archaea 140 663 287 262 380

Bacteria 43 709 197 970 148 550 98 862 152 440

Eukaryota 3 754 227 153 122 207

Virus 593 4 156 6 645 1 151 2 955

**Proportion of unaligned reads (TPM-RSEM) associated to ncRNA*****

FlavoRNA 1 852 372 601 490 052 440 640 623 385

RNaseP 8 544 54 561 44 379 32 797 37 852

tmRNA 11 459 78 416 37 319 15 718 38 519

tRNA, rRNA 257 723 221 946 244 781 76 765

Bacteria 161201

Eukaryota 725329

Others 43 419 33 683 50 669 165 667 67 497

OB: *Original Biofilm*

* The paired reads were aligned to a concatenated sequence consisting of the genomes of strain JAM1 and NL23, and the genome and plasmids of strain GP59.

**Reads that did not align were *de novo* assembled, and the estimation of the relative transcript abundance of the resulting contig was determined and expressed as transcripts per million (TPM-RSEM). The contigs were annotated to find ORFs with their putative function. TPM-RSEM associated to respective ORFs were added by lineage.

*** The contigs were also annotated to find noncoding RNA (ncRNA) with their putative function, and TPM-RSEM associated to them were added. In the OB sample, TPM-RSEM affiliations to Eukaryota and bacterial rRNA were provided. Very low proportions of Eukaryota rRNA were found in the *RR biofilm* samples.

**Table S2. Affiliation of contigs with ORF with relative transcript levels (TPM-RSEM)**

OB C1-1 C4-1 C6-1 C8-1

*Bacteroidota*

*Bacteroidales*

*Lentimicrobium*  9 1070 1448 3619 1334

*Marinilabiliales*

*Geofilum*  1 4357 2348 666 1374

*Mariniphaga*  3 372 2166 646 244

*Sunxiuqinia*  15 1997 2285 3794 886

*Flavobacteriales*

*Aequorivita*  50 1751 3688 450 13926

*Muricauda*  40 67328 5571 9270 21697

*Pseudomonadota; Alphaproteobacteria*

*Hyphomicrobiales*

*Cohaesibacter*  49 579 122 1598 27

other *Hyphomicrobi*um 184 629 1787 952 9308

*Stappia*  16 7658 8866 11093 15445

*Rhodobacterales*

*Paracoccus*  17 2963 5425 2972 3518

*Roseovarius*  113 736 520 922 485

*Rhodospirillales*

*Oceanibaculum*  356 12262 8644 6617 29377

*Pseudomonadota; Gammaproteobacteria*

*Alteromonadales*

*Idiomarina*  28 20553 3903 1634 1915

*Chromatiales*

*Sedimenticola*  2159 7 7 7 5

*incertae sedis*

*Dechloromarinus*  1038 20 31 17 47

*Oceanospirillales*

*Marinicella*  26 2015 10042 174 751

*Pseudomonadales*

*Marinobacter*  100 21243 27004 10848 4753

*Thiotrichales*

other *Methylophaga*  12608 340 366 981 473

*Cycloclasticus*  693 5 3 0.5 2

*Deltaproteobacteria; Bradymonadales*

*Bradymonas*  914 6973 3152 624 2143

*Spirochaetota; Spirochaetal*es

*Spirochaeta*  12 4077 4312 2803 2404

*Chloroflexota*

*Cand. Promineofilum* 799 455 964 1707 1086

*Cyanobacteriota; Leptolyngbyales*

*Leptolyngbya*  1828 64 67 55 269

*Mycoplasmatota*

*Tenericutes*  0 5670 2818 1382 596

*Thermodesulfobacteriota; Desulfovibrionales*

*Pseudodesulfovibrio*  3 1664 509 2208 450

Others (67) 4800 10445 11301 10332 11288

Reads that did not align were *de novo* assembled, and the estimation of the relative transcript abundance of the resulting contig was determined and expressed as transcripts per million (TPM-RSEM). The contigs were annotated to find ORFs with their putative function. TPM-RSEM associated to respective ORFs were added by lineage. Values of these summation are illustrated in this table. Others:TPM-RSEM summation of the 67 other taxa (SupData #4).
